# A Challenging Diagnosis of Febrile Pancytopenia in a Patient With a History of Autoimmune Disease

**DOI:** 10.7759/cureus.35956

**Published:** 2023-03-09

**Authors:** Nehemias A Guevara, Marjorie Mailing Flores Chang, Jorge Castelar, Harry Sequeira, Judith Berger

**Affiliations:** 1 Medicine, St. Barnabas Hospital Health System, Bronx, USA; 2 Medicine/Infectious Diseases, St. Barnabas Hospital Health System, Bronx, USA

**Keywords:** pancytopenia, ciprofloxacin induced pancytopenia, mimic disease, sle initial diagnosis, human brucellosis, serological cross-reactivity

## Abstract

Pancytopenia is a hematologic condition characterized by a decrease in all three peripheral blood cell lines. There are many causes of pancytopenia, and the proper approach is required for accurate diagnosis. Brucellosis and systemic lupus erythematosus (SLE) are both diseases that can initially present as pancytopenia, both of which require a targeted workup to diagnose.

Due to the immune system's complexity, many distinct diseases may have similar symptomatology. Furthermore, infections and rheumatological diseases can stimulate the same molecular pathways and trigger T and B cells. This creates a cross-reactivity between microbial peptides and self-peptides, allowing the spread of microbial-specific T cells that can also respond to self-peptides.

Brucellosis has broad clinical manifestations, often mimicking many other diseases, such as rheumatoid arthritis, sarcoidosis, and SLE. In addition, brucellosis-induced autoantibody production has been described as a triggering factor for immunologic reactions, elevating rheumatological markers by a poorly understood mechanism. Finally, SLE is a well-known medical condition that can mimic several medical conditions, including brucellosis.

We present a case of a young patient who was admitted with febrile pancytopenia. The patient also had IgM antibodies positive for brucellosis and high immune markers for SLE. She was treated for both diseases, and afterward, in retrospect, it was confirmed that the patient did not have acute brucellosis.

## Introduction

Pancytopenia is not a disease but a manifestation of other underlying conditions associated with multiple benign and malignant conditions [1]. Therefore, anyone with pancytopenia needs a relevant workup to identify the underlying etiology [2]. The common causes of pancytopenia vary with age, sex, and geographical area [1,2]. Isolated cytopenias, such as leukopenia, thrombocytopenia, or anemia, are hallmarks of systemic lupus erythematosus (SLE) [3,4].

SLE primarily affects young women. Diagnosis is made by the presence of several antibodies along with clinical symptoms, which are also used to establish the progression and severity of the disease. Nevertheless, several of these markers lack specificity in diagnosing SLE. For example, in a retrospective chart review study, it was found that antinuclear antibodies (ANA) had a sensitivity of 100% but a specificity of 86% for SLE, with a positive predictive value of only 11% [5].

Brucellosis is an uncommon infection transmitted by consuming infected food products from an infected animal (commonly cattle, goats, or pigs) or by handling infected tissue or fluids [6]. The incidence of brucellosis in the United States is estimated to be between 100 and 200 cases per year [7]. Diagnosing brucella infections has proven to be challenging because of its nonspecific manifestations. However, more sensitive and specific diagnostic tools are now available for use. Serological testing is currently used for the diagnosis of brucellosis. However, one such test, standard tube agglutination (SAT), with sensitivity and specificity as high as 95% and 100%, respectively [6], is yet to be readily available. This leads us to use other serological diagnostics, such as enzyme-linked immunoassay (ELISA) or blood cultures.

ELISA is known to cross-react with the following: *Salmonella*, *Francisella*, *Vibrio cholerae*, *Yersinia enterocolitica*, *Serratia marcescens*, *Haemophilus influenzae*, *Pseudomonas aeruginosa*, group A beta-hemolytic *Streptococci*, *Escherichia coli*, and the malaria parasite [8]. These cross-reactions make the interpretation of positive or negative results challenging. In addition, some sporadic cases of positive IgM have been linked to autoimmune diseases like systemic erythematous lupus [9].

If clinical findings fail to differentiate, serology will; they are completely different groups of diseases, but it is reasonable to keep them both on a differential diagnosis in patients with nonspecific findings. Therefore, a case report of a patient with febrile pancytopenia is presented in the following sections of this article, with the challenges of positive tests for SLE and brucellosis.

## Case presentation

A 34-year-old female with a past medical history of sickle cell trait, asthma, and alopecia areata presented to the emergency department (ED) for low-grade fevers, non-productive cough, and diarrhea for two weeks as well as protracted symptoms of fatigue, anorexia, and weight loss. She was originally from the Dominican Republic, and she traveled to her grandmother's farm twice a year. She denied consumption of unpasteurized products or poorly cooked meats. She had a dog and a cat. Family history was remarkable for SLE in two sisters. She did not receive any COVID vaccine. The patient denies malar rash, arthralgia, arthritis, and the Raynaud phenomenon. A review of the system was remarkable only for intermittent headaches.

Vitals signs in ED were blood pressure of 95/64 mmHg; respiratory rate of 18 breaths per minute; heart rate of 107 beats per minute; a temperature of 103.8 degrees Fahrenheit; and oxygen saturation of 98% on room air. Physical examination was remarkable for frontotemporal alopecia, petechiae in the soft palate, and left subclavian lymphadenopathy; her lungs were clear to auscultation, her abdomen was soft and non-tender to palpation, and she did not have lower extremity edema.

Initial blood work was remarkable for pancytopenia, elevated transaminases, and urine proteinuria of 100 mg/dL (Table 1). Computerized tomography of the abdomen and pelvis showed splenomegaly with lymphadenopathy adjacent to the splenic hilus and a prominent posterior periportal lymph node. Cefepime and doxycycline were initiated by infectious disease recommendation leading to a cessation of fevers but the development of new inguinal lymphadenopathy.

**Table 1:** Initial laboratory data MVC: Mean corpuscular volume; MCH: Mean corpuscular hemoglobin; MCHC: Mean corpuscular hemoglobin concentration; TSH: Thyroid stimulant hormone; LDH: Lactate dehydrogenase; ESR: Erythrosedimentation rate; ALT: Alanine transaminase; AST: Aspartate transaminase.

Variables	On admission	Reference range
White cell count	1.4	4.2–9.1 10^3^/uL
Neutrophils (%)	47.1%	34.0–67.9%
Neutrophils (10^3^/uL)	0.66	1.56–6.13 10^3^/uL
Lymphocytes	47.9%	21.8–53.1%
Monocytes	4.3%	5.3–12.2%
Eosinophils	0.0%	0.8–7.0%
Hemoglobin	8.8	13.7–17.5 mg/dL
Hematocrit	25.1	40.1–51.0%
Platelet count	89	150–450 10^3^/uL
MCV	83.9	79.0–92.2 fL
MCH	29.4	25.7–32.2 pg
MCHC	35.1	32.3–36.5 mg/dL
Sodium	127	135–145 mEq/L
Potassium	4.0	3.5–5.3 mEq/L
Chloride	101	96–108 mEq/L
Glucose	111	70–99 mg/dL
Calcium	8.6	9.2–11.0 mg/dL
Creatinine	0.7	0.6–1.2 mg/dL
ALT	334	4–36 IU/L
AST	420	8–33 IU/L
Bilirubin total	1.0	0.1–1.2 mg/dL
Alkaline phosphatase	166	38–126 IU/L
TSH	0.50	0.34–5.60 u(IU)/mL
Magnesium	1.9	1.3–2.1 mEq/L
Troponin	<0.10	0.00–0.48 ng/mL
LDH	757	100–190 IU/L
ESR	15	0–20 mm/hr
C-reactive protein	0.21	0.00–1.00 mg/dL
Procalcitonin	0.04	0.00–0.08 ng/mL
Urine analysis		
Color	Yellow	
Clarity	Clear	
Gravity	1.011	1.003-1.035
pH	5.0	5.0-8.0 pH units
Protein	100	Negative mg/dL
Glucose	Negative	Negative mg/dL
Bilirubin	Negative	Negative/Positive
Blood	Small	Small, Moderate, Large
WBC	6	0-5
RBC	3	0-2
Squamous epithelial cells	3	0-4
Bacteria	Few	None, few
Mucus threads	Rare	None
Renal epithelial cells	<1	<1
Granular cast	<1	0-0
Cellular cast	1	0-0

An extensive workup for infectious, hematological, and nutritional etiologies of her initial presentation was also sent (Table 2), which was remarkable for the positive IgM brucella antibody, high titers of ANA, DNA double-stranded (dsDNA) antibody, low complement C4 (3 mg/dL), and borderline complement C3 (92 mg/dL).

**Table 2:** Complementary laboratory data

Variables	Results	Reference range
Antinuclear antibodies (ANA)	Positive – 1:2560	• Negative: <1:80
		• Borderline: 1:80
		• Positive: >1:80
Anti-dsDNA	Positive – 129	0–9 IU/mL
B12	769	160–950 pg/mL
Folic acid	6.7	3.0–999 ng/mL
Rickettsia	Negative < 1.64	<1:64 – Negative
Typhus	Negative	• Negative: <1:64
		• Present or past: 1:64
		• Recent/active: >1:64
Rocky Mountain spotted fever IgG	Positive >1:64	<1:64 – Negative
A) Brucella IgG antibody	Negative	Negative or positive
B) Brucella IgM antibody	Positive	Negative or positive
C) Brucella IgG antibody	Negative	Negative or positive
D) Brucella IgM antibody	Negative	Negative or positive
Infectious mononucleosis	Negative	Negative or positive
Strongyloides IgG antibody	Negative	Negative or positive
Human immunodeficiency virus (HIV)	Non-reactive	Reactive or non-reactive
Blood culture	No growth	
Urine culture	No growth	
Stool culture	Negative for Salmonella, Shigella, Campylobacter, and Yersinia. Negative for Escherichia coli serogroup 0157/enterohemorrhagic E. coli	
COVID PCR test – nasopharyngeal swab	Negative	

The bone marrow aspiration and biopsy were within normal limits (Table 3) based on the European Alliance of Associations for Rheumatology/American College of Rheumatology (EULAR/ACR).

**Table 3:** Bone marrow studies AML: Acute myeloid leukemia; NHL: Non-Hodgkins lymphoma.

Variables	Results
Bone marrow aspirate	There is a mixed population of maturing myeloid cells, B cells, and T cells. No abnormal myeloid maturation is seen. CD14+ monocytes are 3% of total cells. There is no increase in CD34 positive blasts, comprising <1% of the total cells. The B cells (4% of the total) are polytypic, and the T cells (9% of the total) show no pan T cell antigen deletion. A monoclonal plasma cell (CD38 bright) population is not identified.
Bone marrow biopsy	Bone marrow, right posterior superior iliac bone, biopsy:
	-Normocellular marrow (60%-65%), with trilineage hematopoiesis with maturation.
	-No tumor or vasculitis is seen in the sections examined.
	-Concurrent flow cytometry is reported as there is no evidence of abnormal myeloid maturation or an increased blast population. There is no evidence of a lymphoproliferative disorder or plasma cell neoplasm.
	-Fluorescence in-situ hybridization (FISH): AML panel: negative; NHL probes: negative.

The patient met 14 SLE criteria to be diagnosed with SLE. Therefore, a diagnosis of SLE flare with febrile neutropenia was made. The patient was started on steroids and hydroxychloroquine. A repeated test for brucella was done due to suspected cross-reactivity in the setting of an acute lupus flare, which was negative (item D in Table 2). After discharge, she followed up with rheumatology at an outpatient clinic with complete resolution of symptoms and progressive return of the pancytopenia at baseline, with just persistent mild normocytic normochromic anemia.

## Discussion

SLE is a chronic, systemic autoimmune disease characterized by inflammation and organ damage. Disease mechanisms are loss of self-tolerance to antigens due to autoreactive B cells with downstream development of autoantibodies. In addition, the body develops an immune response against autologous nucleic acids [10]. Infectious processes and SLE share many similarities; infections trigger an immune response that protects the body against them, and SLE activates an autoimmune reaction to establish organ damage. In addition, it has been documented that viruses, bacteria, and protozoa can cause immune dysfunction through molecular mimicry [11,12] and epitope spreading.

The immune system’s complexity has led to the study of cross-reactivity of diseases and SLE as a prototype of autoimmunity. T and B cells are triggered by infection as molecular mimicry because of the structural similarity between microbial peptides and self-antigens [13]. This creates a cross-reactivity with microbial peptides and self-peptides, allowing the spreading of microbial-specific T cells that can also respond to self-peptides [14]. This mechanism allows for the development of superantigens that can bind to the variable domains of T cell receptors and major histocompatibility complex class II. This entails developing many T cells with different antigenic specificities and downstream activation of autoimmune reactions. The presentation of self-antigens by an antigen-presenting cell (APC) causes the spreading of T cells by epitope, creating an overprocessing and overrepresentation of self-antigens. In addition, as T cells are activated, cytokine production increases autoreactive or memory T cell expansion via bystander activation.

Subsequently, the impaired clearance of apoptotic cells and the resultant nuclear materials exposed to the immune system increase the production of autoantibodies or activate autoreactive lymphocytes [15,16]. This creates a vicious cycle that allows for cross-reactivity with autoimmune diseases. This mechanism has been described as a trigger of autoimmunity. Moreover, bacteria and viruses act on the intracellular toll-like receptors through intracellular signaling pathways, stimulating the type-I interferons (type-I IFNs) and, in particular, IFN-alpha, which is a major step in the pathophysiology of SLE [17]. Furthermore, several cases that describe SLE mimicking infections and vice versa have been documented.

Our patient had IgM positive for brucella, which is a gram-negative, catalase- and oxidase-positive, unencapsulated, facultative intracellular coccobacillus [18,19]. It is a systemic infection that may involve any organ [20]. Clinical characteristics are broad, and it has different stages such as acute, sub-acute, chronic, relapsing, active, and non-active. Signs and symptoms include chills, stiffness, anorexia, weakness, weight loss, headache, night sweats, arthralgia, arthritis, myalgia, dizziness, dyspnea, hepatomegaly, splenomegaly, and mouth ulcerations [7,20-27]. Given these broad clinical manifestations, SLE is one of the differential diagnoses that must be considered with such a presentation. In addition, brucellosis has hematologic manifestations such as leukopenia, anemia, thrombocytopenia, and pancytopenia [27]. Therefore, inflammatory markers have been proposed as helpful tools to differentiate between rheumatologic diseases and brucellosis.

The diagnosis of brucellosis can be made by culture, serological testing, or nucleic acid amplification assays. Diagnosis is not straightforward because there is no single test for defining the disease against which all laboratory assays should be measured, given that the manifestations of brucellosis are broad and nonspecific [28,29]. The definitive diagnosis of brucellosis can be made with the isolation and culture of brucellae from sterile body fluids or tissues with a specificity of 100%. Still, the sensitivity of cultures decreases with the progression of the infection [28]. On the other hand, nucleic acid amplification assays (PCR) have, in general, an unmatched sensitivity [30]. Brucella’s PCR tests show disagreement among laboratories and no standardization of critical technical aspects [30]. Moreover, it has been demonstrated that seven out of 10 patients continued to exhibit positive PCR results from 24 to 36 months after antibiotic therapy, despite the absence of symptoms indicative of persisting disease or relapse [31]. Furthermore, PCR does not discriminate between viable and dead organisms and must be interpreted based on the absence or presence of ongoing symptoms [28].

Since no gold standard for diagnosis exists, serodiagnostic tests for brucellosis are frequently evaluated by comparing results with those obtained with other serological assays, used alone or in combination [24]. Despite their numerous drawbacks, serological tests remain a cornerstone in diagnosing brucellosis in underdeveloped and first-world countries [29]. In addition, brucellosis can act as a stimulating immunological reaction mediated by multiple antibodies [32], which seems to cause positive autoimmune tests due to immunological responses [33].

The humoral immune response has been studied, and there is an increase in all types of IgM, IgG, C3, and C4 for a patient with acute brucellosis, and low titers of ANA with rheumatoid factors have been found in 25% and 37% respectively. Furthermore, immune complexes were positive in 91.5% of a sample of 300 patients [33,34]. Several studies have demonstrated the ability of brucellosis to produce ANA positivity. Given its significant variability, the ANA titer is essential when diagnosing SLE and starting a specific treatment. For example, ANA could be positive in 25%-30% of healthy populations, but the rate decreases to 5% with an increase in ANA titer from 1:40 to 1:160 [31]. Anti-dsDNA is more specific for SLE and can be positive in more than 70% of SLE patients, while only 0.5% of patients were without the disease. However, serology has to be interpreted along with the clinical symptoms [33]. Therefore, ANA presence in an elderly patient with negative dsDNA could be explained by age. On the other hand, positive ANA in a young female with positive dsDNA should be considered SLE [33]. 

Our patient presented with high titers of ANA and a strong positive titer for dsDNA; furthermore, the patient met 14 SLE criteria of the European Alliance of Associations for Rheumatology/American College of Rheumatology (EULAR/ACR). The diagnosis of SLE was challenging because of the infection with cross-reactivity and positive serum markers of brucellosis. Even more, fever and white blood cell count/neutrophils improved with antibiotics, indicative of concomitant infection. Given the cross-reactivity of SLE with infections and the overlapping pathways with several diseases, procalcitonin (PCT) and C-reactive protein (CRP) have been studied to aid in the differentiation. A retrospective study with 177 patients measuring PCT and CRP found that CRP was an effective marker for diagnosing infection in SLE patients [35]. However, other studies have demonstrated that PCT has a negative predictive value for bacterial infection in active SLE [36]. Our patient had normal CRP and PCT, making an infectious process less likely. In addition, IgM, IgG, and cultures were negative for brucellosis in the follow-up period.

## Conclusions

A proper clinical assessment and laboratory workup for an infectious process should be made when dealing with a patient with febrile neutropenia; differential diagnoses are broad, and most of the time, an extensive workup is required and pertinent. Even if the clinically autoimmune disease is the most likely diagnosis, other options need to be ruled out to provide proper treatment. We also have to remember that an autoimmune disease can cause cross-reactivity with other antibodies without true infection, but having an autoimmune disease does not mean that a patient cannot coexist with ongoing infections.
